# Insect Epigenetic Mechanisms Facing Anthropogenic-Derived Contamination, an Overview

**DOI:** 10.3390/insects12090780

**Published:** 2021-08-31

**Authors:** Gabriela Olivares-Castro, Lizethly Cáceres-Jensen, Carlos Guerrero-Bosagna, Cristian Villagra

**Affiliations:** 1 Instituto de Entomología, Universidad Metropolitana de Ciencias de la Educación, Avenida José Pedro Alessandri 774, Santiago 7760197, Chile; cristian.villagra@umce.cl; 2 Laboratorio de Físicoquímica Analítica, Departamento de Química, Facultad de Ciencias Básicas, Universidad Metropolitana de Ciencias de la Educación, Santiago 7760197, Chile; lyzethly.caceres@umce.cl; 3 Department of Physics, Chemistry and Biology (IFM), Linköping University, 581 83 Linköping, Sweden; carlos.guerrero.bosagna@liu.se; 4 Environmental Toxicology Program, Department of Integrative Biology, Uppsala University, 752 36 Uppsala, Sweden

**Keywords:** imidacloprid, insectageddon, hormetic responses, sublethal exposure

## Abstract

**Simple Summary:**

Epigenetic molecular mechanisms (EMMs) are capable of regulating and stabilizing a wide range of living cell processes without altering its DNA sequence. EMMs can be triggered by environmental inputs. In insects, EMMs contribute to explaining both negative effects as well as adaptive responses towards environmental cues. Among these stimuli are chemical stressors, such as pesticides. We review the link between EMMs and pesticides in insects. We suggest that pesticide chemical behavior promotes both lethal and sublethal exposure of both target and non-target insects. As a consequence, for several native and beneficial insect (e.g., pollinators), EMMs are involved in diseases and disruptive responses due to pesticides, while in the case of pest species, EMMs are linked in the development of pesticide resistance and hormesis. We discuss the consequences of these in the context of insect global decline and biotic homogenization.

**Abstract:**

Currently, the human species has been recognized as the primary species responsible for Earth’s biodiversity decline. Contamination by different chemical compounds, such as pesticides, is among the main causes of population decreases and species extinction. Insects are key for ecosystem maintenance; unfortunately, their populations are being drastically affected by human-derived disturbances. Pesticides, applied in agricultural and urban environments, are capable of polluting soil and water sources, reaching non-target organisms (native and introduced). Pesticides alter insect’s development, physiology, and inheritance. Recently, a link between pesticide effects on insects and their epigenetic molecular mechanisms (EMMs) has been demonstrated. EMMs are capable of regulating gene expression without modifying genetic sequences, resulting in the expression of different stress responses as well as compensatory mechanisms. In this work, we review the main anthropogenic contaminants capable of affecting insect biology and of triggering EMMs. EMMs are involved in the development of several diseases in native insects affected by pesticides (e.g., anomalous teratogenic reactions). Additionally, EMMs also may allow for the survival of some species (mainly pests) under contamination-derived habitats; this may lead to biodiversity decline and further biotic homogenization. We illustrate these patterns by reviewing the effect of neonicotinoid insecticides, insect EMMs, and their ecological consequences.

## 1. Introduction

Epigenetics is a complex field of research concerning the molecular mechanisms capable of modifying DNA expression through organism’ ontogeny. These changes can even become inheritable [1]. Epigenetic alterations can take place through an ever-expanding set of chemical modifications, known as epigenetic molecular mechanisms (EMMs), including changes affecting nucleotides such as DNA methylation (i.e., C5-cytosine methylation), post-translational modifications of histone variants, and nucleosome configuration, as well as noncoding RNA effects on living cells, among others [1,2,3] (Table 1). Epigenetic changes can take place stochastically, although EMMs are often the result of the influence of environmental cues on organisms [4]. Epigenetics has gained increasing interest among basic and applied scientists, originating a mounting body of interdisciplinary research and providing us with novel approaches to longstanding questions in biological sciences [1,5,6]. Several insect species have contributed as model organisms to the study of EMMs. These processes have been found to contribute in the regulation and stabilization of the basic self-organizing functions of insect life. Among these are the response of insects towards different environmental cues and disturbances, including those derived from human activities. Therefore, EMMs participate in the insect expression of varied responses to environmental stimuli such as polymorphisms and social caste specialization [7,8,9], insecticide resistance [10,11], and a myriad of other aspects of insect biology in model systems and wild species [12,13]. Unfortunately, while in recent decades we have been able to enrich current explanations of EMMs thanks to the study of varied aspects of insect life in several species, at the same time, their populations have been experiencing steady declines [14,15,16].

Insect species reduction in diversity and population abundance has been accompanied by the demise of several other associated organisms (including symbionts), in what has been called the sixth mass extinction [28,29,30,31,32,33]. Several factors contributing to biodiversity loss are derived from the application of technological packages associated with human activities such as extractive and industrial production as well as industrialized intensive agriculture [34]. The contamination from chemicals incorporated during agricultural field management (e.g., pesticides and fertilizers) has been found to be one of the most harmful to the environment and human health [35,36,37]. These alterations increase the threats to a wide range of organisms, from beneficial soil microorganisms to our own species [38,39,40]. Pesticides can cause chronic adverse health effects due to altered gene expression mediated by EMM, e.g., C5-cytosine methylation (hereafter “DNAm”) [35]. Several negative effects of sub-lethal doses of pesticides have been observed in the western honeybee, *Apis mellifera* (Hymenoptera: Apidae), which include reduced fecundity of queens; impaired immune response; flawed navigation and learning ability; and physiological, reproductive, and developmental negative side effects [41,42]. Pesticides have also been linked to DNAm alterations in bees [41]. Similar perturbations of biological processes have been observed in ground-nesting and solitary bees, with decreases in brood production and larval hatching as well as impaired foraging behavior [43]. EMMs have been proposed to provide resilience to environmentally derived stresses, including pesticides. It has been demonstrated that insects may be capable of developing resistance to anthropogenic chemical contaminants thanks to epigenetic modifications [44,45,46]. This has been found in several pest insects, where being exposed to these chemical formulations allows these pests to tolerate other sources of stresses [44]. This suggests that anthropogenic stress not only may be directly harmful to native insects but also may promote the rapid evolution of resistance in pest species. As a consequence, biodiversity and the further homogenization of industrial agriculturally managed land have seen steady declines [47].

Here, we review the current knowledge about EMMs in insect biology considering the epigenetic effects of different anthropogenic contaminants, with special emphasis on pesticides. We review in detail the case of the neuro-active neonicotinoid insecticides in non-target insect species and their relation to the decline in managed and wild bee species, drawing a potential link between EMMs and pesticide contamination and explaining colony collapse disorder [41,48]. Finally, we discuss the relationship between EMM responses to anthropogenic contamination and their potential role in the context of the sixth mass extinction [32].

## 2. Epigenetic Molecular Mechanisms

**Figure 1:**
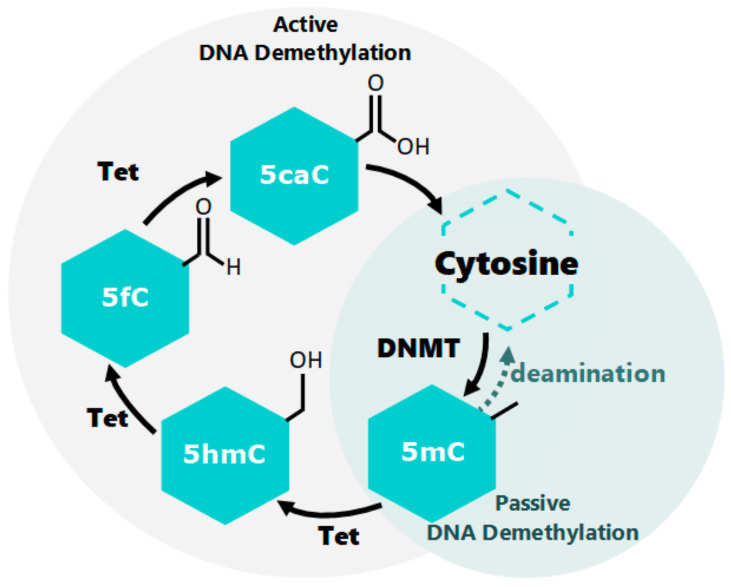
DNA demethylation and methylation: DNAm in eukaryotes occurs mainly at cytosine’s fifth carbon atom, catalyzed by de novo methyltransferases (*DNMT*). Active DNA demethylation involves the successive oxidation of the 5-methyl-cytosine *(5-mC)* to 5-hydroxymethyl-cytosine *(5-hmC),* to 5-formyl-cytosine *(5-fC)*, and then to *5-carboxy- cytosine (5-caC)*, catalyzed by the Ten-eleven translocation enzyme family *(Tet).* Passive DNA demethylation occurs during replication due to the absence of methylation by DNMT1 across several rounds of replication. Figure by Gabriela Olivares-Castro [59,60].

**Figure 2:**
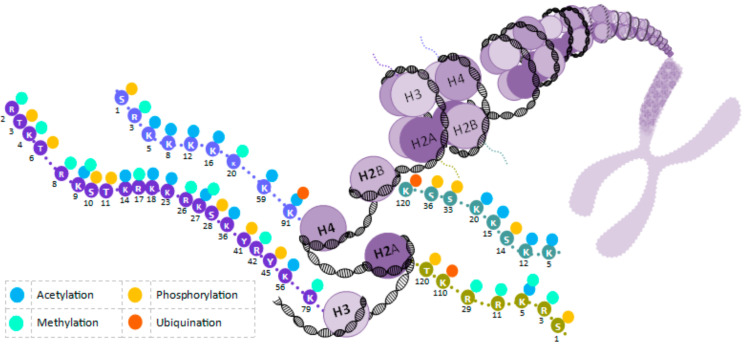
Histone modifications: Histones are proteins that package DNA into nucleosomes. Every nucleosome contains two subunits each of histones H2A, H2B, H3, or H4. These histones have an N-terminal tail where the covalent modifications preferentially occur. These modifications can be acetylation/deacetylation, methylation, phosphorylation, or ubiquinitation. All of these modifications can be related to different effects on the biology of the organism. Histone acetylation is triggered by the addition of an acetyl group and is involved in various cell processes such as chromatin dynamics and transcription, and apoptosis, among others. Histone methylation occurs via the addition of a methyl group and causes transcription repression or activation, depending on target sites. Histone phosphorylation happens when a phosphate group is added to the histone; it is involved in DNA repair, cell cycle progression, chromosome condensation, and apoptosis. Histone ubiquitination happens with the transport of ubiquitin to the histone core proteins and can either activate or inhibit target gene expression, depending on which histone is affected. Figure by Gabriela Olivares-Castro [61].

**Figure 3:**
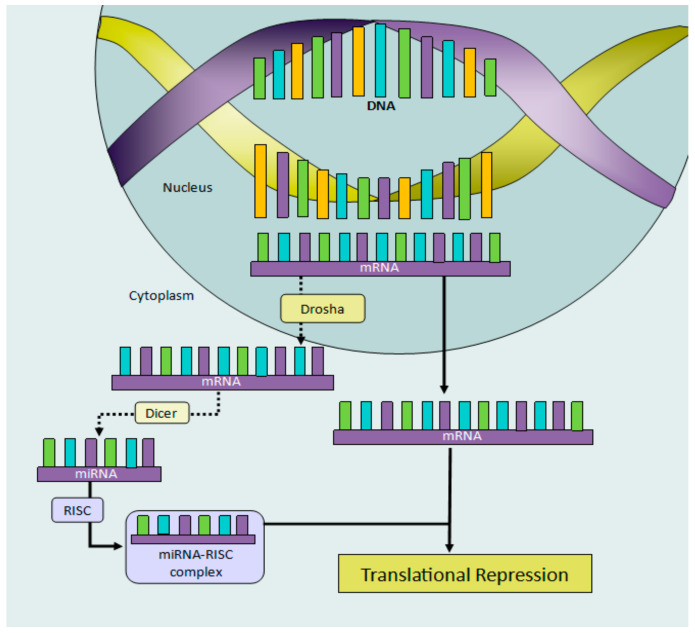
miRNA translational repression: to block protein expression, miRNA target mRNA and bind to them, preventing translation. Inside the nucleus, primary miRNA are cleaved by RNase III Drosha to produce smaller precursors of 60–70 nucleotides, which then translocate to the cytoplasm. Once there, the precursors are processed by another RNase III Dicer, which generates a duplex RNA of ~22 nucleotides. One strand is degraded, and the other is incorporated into the RNA-induced silencing complex (RISC), where it binds to the 3′ untranslated region of complementary mRNA. As a consequence, this may result either in its degradation and/or reduced translation, which has been proposed to lead to translational repression. Figure by Gabriela Olivares-Castro [62].

As previously mentioned, epigenetics focuses on the study of molecular interactions that modify gene expression both during cells’ differentiation as well as later in the life of an organism, during cells’ quiescence [49]. Epigenetic patterns are mainly defined as the result of the interaction of individuals with their surrounding environment [50]. EMMs include many processes involving biochemical reactions, with the collaboration of different biomolecules in living organisms capable of generating transient or heritable changes in gene expression [1,51]. A broad distinction can be made between pre- and post-transcriptional epigenetic mechanisms, depending on the levels in which they act upon gene expression. The former chemically modifies DNA nucleotides or the tails of histone proteins that wrap DNA around the nucleosome to form chromatin [6]. The most studied EMMs are DNAm (Figure 1), histone acetylation/deacetylation, histone methylation, histone phosphorylation, and histone ubiquitination (Figure 2) [52,53]. In contrast, a large number of known post-transcriptional mechanisms are associated with RNA, including transcripts, messenger (mRNAs), and non-coding (miRNAs, sRNAs, and lncRNA) [54,55,56,57,58]. Figure 3 shows miRNA transcription repression.

**Figure 4:**
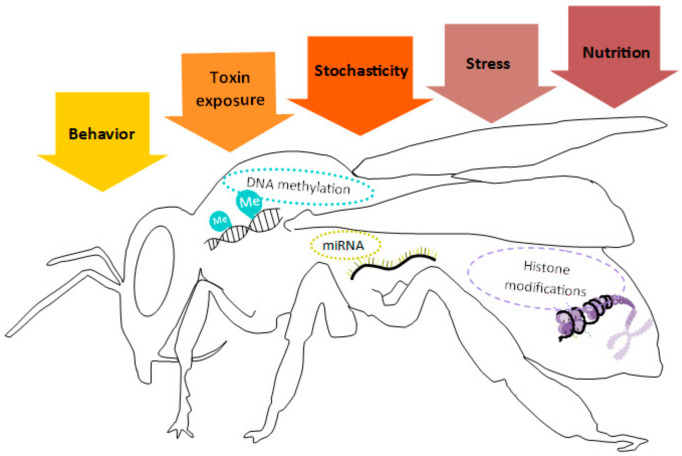
Environmental factors that trigger epigenetic mechanisms: Schematic representation of an insect (represented by a bee silhouette) illustrating the main EMMs found. The colored arrows denote the five main environmental factors that have been described in the literature that trigger the different epigenetic responses in the organism. These responses may be pre-transcriptional such as DNAm and histone modifications, or post-transcriptional such as miRNA interference. Figure by Gabriela Olivares-Castro [64].

The epigenome comprises all of the epigenetic modifications associated with a given genome in the form of chemical modifications of DNA and histone tails [63]. There are five environmental factors that define the origin and maintenance of epigenome modifications: behavior, stress (both biotic and abiotic), nutrition, toxin exposure, and stochasticity (in the placement of methylation marks). All of these factors are especially relevant during early developmental stages [64]. Figure 4 shows the main factors reported to be associated with EMMs in insects: DNAm, histone modifications, and micro-RNA (miRNA) interference.

Recent insightful reviews of EMMs illustrate current advances in the study of epigenetic modifications in insects [2,7,65,66]. It has been found that EMMs generally allow for the development of biological responses to environmental challenges, including those of anthropogenic origin. An example of these are EMM-related pesticide resistance, which has been observed from aphids to mosquitoes [45]. EMMs can also play a role in the protection of inheritance. For example, EEMs have been shown to be highly involved in insect transposon silencing through their potential to suppress genome rearrangements [54].

DNA expression changes induced by EMMs can be inherited by the descendants of the exposed individuals through transgenerational epigenetic inheritance, which bypasses the reprogramming of DNAm and chromatin proteins during meiosis [67]. Some authors consider that the heritability of epigenetic changes, either as a cell lineage effect or in a transgenerational sense, is their defining quality [64]. An extensive recent review on the subject of epigenetic inheritance by Tikhodeyev [68] describes ten different mechanisms of stable allelic epigenetic inheritance (SAEI) and discusses two other possible mechanisms. In Table 1, we provide an updated version of Tikhodeyev’s summary of different mechanisms of epigenetic inheritance, in which we incorporate recent examples of SAEI, focusing on the three main epigenetic mechanisms discussed in this work.

### Insect Epigenetics and Contamination

Genetics has benefited greatly from the study of various insect models (class *Insecta*), which have been used in the discovery and characterization of different molecular mechanisms [66,69,70,71,72,73,74,75]. Major topics in biology have been enriched by the feedback from the study of insect EMMs, such as cell biology, development, and evolution [54]. For example, epigenetics has contributed to enriching explanations regarding the mechanisms underlying polymorphisms such as sociality in insects, where EMMs have been shown to be relevant in explaining caste differentiation [76,77]. EMMs have also contributed to explaining how environmental influences alter different aspects of phenotypes without modifying genotypes [78]. 

The study of anthropogenically derived influences as epigenetic stimuli, such as pollution and climate change, are among the fast-growing areas of interest within insect EMMs [79,80,81]. The goal of these studies is to assess how stressors may affect different ecological levels [82], considering exposure to pesticides [41,44,83], endocrine disruptors [11,84,85], heavy metals [84,86,87], or temperature changes [88] as triggers of epigenetic changes. In Table 2, we summarize the different responses of insect biology to different kinds of toxicants and whether there is evidence of the role of EMMs in these processes.

Besides direct lethality, a wide array of sub-lethal effects has been described in insect biology in response to different levels of exposure to environmental toxicants [134,135,136,137,138]. Population hormetic responses appear after an organism is stimulated by a relatively low dose of a stressor; thus, this may enhance their chances of survival under the pressure of this environmental stress source [125]. For example, exposure to sub-lethal pesticide traces can induce changes such as a reduction in sucrose sensitivity [121], a deformation of the Malpighian tubules [120], or the development of resistance towards the pesticide [102,105,123]. Although some of these effects have been linked to EMMs by previous researchers [54,120], there are other responses towards different pesticides where it is possible to hypothesize the involvement of EMMs [83,139], for instance, the activation of the cytochrome P_450_ monooxygenase (CYP450) pathway after exposure to a low dose of pesticide [83]. 

Over half of the studies shown in Table 2 were conducted on dipterans as model organisms, with considerable focus on species with aquatic phases during development such as mosquitoes (e.g., [11,84,132]). Of all the toxicants reviewed, the only group without studies in aquatic environments is the carbamate cholinesterase inhibitors, a group of organic compounds derived from carbamic acid (NH_2_COOH) found in broad-spectrum pesticides (e.g., [107,108,109,110]). Despite the concern raised about carbamides and their effects on human health [36,140], this group is one of the least studied regarding their effect on biodiversity, including arthropods [141]. Carbamates have been found among the cocktail of contaminants detected in irrigation water sources, along with other pesticides, microplastics, and pharmaceutical residues. Importantly, these contaminants may exert synergistic negative effects on aquatic organisms [142]. 

Organophosphates such as diazinon and glyphosate have shown remarkably high sorption and irreversible sorption in allophanic agricultural soils [143,144]. It has been reported that glyphosate has consequences at the ecosystem level, affecting the soil and water sources due to its leaching from the soil [143]. Evidence also shows glyphosate teratogenicity in vertebrates including our own species, reporting unwanted effects in non-target organisms for GPS ranging from physiological abnormalities to carcinogenesis [143]. GPS also affects the intestinal microorganisms of bees, increasing the mortality of native bees [137]. 

Only 20% of the studied organisms in Table 2 are native species, with none of the studies exploring the involvement of EMM responses to toxicants, contrasting with 40% of studies considering plague organisms and 40% conducted on model species. These values reflect how limited the currently available information is on these kinds of effects in native species regarding pesticides and other toxins, an aspect that should be further explored if loss of biodiversity is to be addressed.

In contrast, endocrine disruptors are the group with the most studies in insects, about 22.4% of reviewed articles, with the majority from aquatic environments, followed closely by organophosphates with 17.2%. The most frequently observed effects in these cases were epigenetic modifications as well as changes in fertility and egg hatching. DNAm was the most frequently observed of the EMMs reviewed in Table 2, being described in dipterans of the culicid and chironomid families as well as in hymenopterans such as honeybees. RNA-based modifications were also described in aquatic dipterans from the culicid and chironomid families, reinforcing the importance of aquatic organisms in toxicological research. It is relevant to highlight that most of the consequences of the contaminants reviewed can be categorized as sub-lethal alterations. This stresses the need to reconsider the protocols broadly used to assess the effects of pesticide toxicants on study organisms for further applied use in agriculture farmlands and urban environments, as sub-lethal exposure is the most likely source of EMM-related alterations, since a “sub-lethal” exposure to contaminants is capable not only of generating detrimental environmentally induced effects in the organisms directly affected but also in their progeny. This may result in pervasive transgenerational alterations to insect populations and species.

Even in the cases where effects on EMMs in response to a specific chemical have not been studied, it is possible to hypothesize their potential involvement in several of the phenotypic alterations detected due to exposure to contaminants (Table 2). This can be achieved through comparing similar effects of contaminants in the focal species previously investigated, where phenotypic alterations (e.g., physiological) had a clear link with EMMs [54]. This approach may help to identify further links between human-derived pollution and EMMs to be explored in future studies. The participation of EMMs as regulators of insect responses to pesticides can also be inferred by analyzing the methylome and genome in specific experimental situations such as resistance to a certain chemical [145].

Different innovative approaches are being employed to address this problem, such as in vitro studies using vertebrate and invertebrate model organisms. Several of these toxicants have been identified as capable of affecting epigenetic states in humans [60,140,146]. In the following section, we delve into the interactions between pesticides and the environmEntomol.

## 3. Pesticide Chemical Behavior in the Environment

Pesticides include many classes of chemicals used to control undesirable target species such as agricultural or household pests [147]. These can be chemical extracts derived from the metabolites of different organisms such as pyrethrins [148] and neem seed oil [149]. However, most pesticides used are synthetic chemical formulations massively produced and distributed by pharmaceutical companies [150]. As has been extensively demonstrated and reviewed, pesticides not only harm the intended target species but also, most of the time, can exert negative effects on a diversity of other non-target life forms exposed to them, including humans [3,151,152,153]. Pesticide formulations have been found to have adverse consequences in the central nervous system; cognitive abilities and behavior; reproduction; endocrine regulation; development; the promotion of diseases (e.g., cancer) [154,155,156,157]; and ultimately the survival of many organisms, including pollinators exposed to these agrochemicals [135,158,159].

The global amount of pesticide application has remained constant during recent decades; nonetheless, it has been reported that the toxicity of these formulations has been increasing. The total worldwide expenditure in pesticides exceeded 56 billion USD in 2012. Herbicides are the dominant pesticide used to control weeds in agricultural production, accounting for the largest proportion (45%) of total pesticide expenditure, followed by insecticides, fungicides, and other pesticides [160].

The main processes studied to evaluate the environmental fate of herbicides are sorption kinetics, sorption–desorption, degradation, biodegradation, bioavailability, and transport [161,162,163]. Sorption is the key parameter in evaluating the fate and behavior of herbicides in soils in relation to bioavailability, distribution, and transport to other environmental compartments [164]. The sorption process in soils is one of the most relevant aspects to consider for appropriate use of herbicides and their environmental fate. The chemical properties of herbicides together with the physical, chemical, and biological characteristics of soils influence their fate and behavior in soils [144].

Volcanic ash-derived soils (VADS) such as andisols generally have a high concentration of total phosphorus but not plant-available P, so these soils require frequent adjustments of the soil pH, replacement of exchangeable Mg, and heavy P applications in order to yield crops [144]. These industrial agriculture amendments can increase the leaching potential of organophosphate herbicides such as glyphosate (GPS) (Table 2) that have been recently applied as a consequence of competition with phosphate for surface sites, an increase in negative charge on the surface resulting from phosphate sorption [165]. These aspects help to explain how pesticide residues can be adsorbed in the soil as well as can infiltrate into groundwater [122,166,167,168,169].

The broad-spectrum herbicide GPS is used non-selectively in agriculture to control weeds and herbaceous plants [143,165]. GPS sorption studies on VADS have reported that it is strongly adsorbed by mineral clays and organic matter (hereafter “OM”) from agricultural VADS [143,165]. GPS has shown irreversible sorption in allophanic agricultural soils [143,165]. The exceptionally high sorption of GPS in ultisols has been related to kaolinite content and acidic *pH* [165].

An excess of GPS sorption in unfertilized VADS may result in an unavailability of this herbicide to targeted pests as well as uneven distribution around the plants [170]; microbial degradation of this herbicide to its primary metabolites, glioxylate and aminomethylphosphonic acid (AMPA) [143,144,165]; alteration of soil microbiota; and adverse effects on multicellular species [171]. It has been reported that GPS has consequences at the ecosystem level, affecting the soil and water due to its leaching from the soil [143]. The contamination of water sources by pesticides may also trigger detrimental effects in several aquatic insects during early development stages [170,172]. Evidence also shows GPS teratogenicity in vertebrates, including our own species, reporting unwanted effects in non-target organisms ranging from physiological abnormalities to carcinogenesis [161]. GPS also affects the intestinal microorganisms of bees, increasing the mortality of native bees [143].

Similar patterns of environmental risks due to pesticide use have been reported for other formulations such as carbamates and neonicotinoid insecticides, which were initially considered not harmful but in which the risks are currently under question [173]. Carbamate insecticides (Table 2) have been found among the cocktail of contaminants detected in irrigation water sources, along with other pesticides, microplastics, and pharmaceutical residues. Importantly, these contaminants may exert synergistic negative effects on aquatic organisms [142].

Neonicotinoids such as acetamiprid (ACT) and imidacloprid (IMD) (Table 2) are insecticides widely used in agriculture [164]; they exert a powerful systemic action on insects for the protection of various crops against piercing and sucking pests, with low acute and chronic toxicity for mammals, birds, and fish [164,174]. Neonicotinoids usually have high water solubility and a low octanol–water partition coefficients (*K_ow_*) [164] and are not readily biodegradable [164]. All neonicotinoid insecticides are rather stable at acidic or neutral *pH* and even at alkaline *pH* (half-life from 11.5 to 420 days) [164], since they hydrolyze slowly. The half-lives of ACT and IMD are 3–8 weeks, and 13 and a half months, respectively [174]. These properties of neonicotinoid insecticides influence their fate in the environment, since between 2 and 20% of the active ingredient is adsorbed by the plant (depending on the type of crop), with an average adsorption of 5%; thus, between 80% and 90%, or even >95% of the bulk active ingredient is spread in the environment and enters the soil or surface/ground water after planting [164,174].

Neonicotinoids easily diffuse into soils and waterways in agricultural settings and their neighboring areas through leaching and runoff from arable lands [174,175] due to their hydrophilic character and low potential for sorption to soil [174], raising concerns that they may pass into bodies of water and therefore can pose a risk for water quality. Neonicotinoids have been found in the aquatic environment in almost all continents at concentrations above the European Union (EU) legislation limits [164].

The amount of neonicotinoid sorption to soil increases with increasing soil *OM* content and a slower sorption rate due the presence of chemical non-equilibrium [161,174,175]. Neocotinoid sorption increases with the aging of soil residues due to sorption kinetic processes and diffusion of this kind of insecticides, which can lead to an unexpected persistence of neocotinoids in the environment, decreasing the potential risk of leaching to deeper layers [175].

It has been reported that the fraction of humic substances (HS) (HA, fulvic acids (FA), and humin (HM)) can be more important in determining neocotinoid sorption parameters given the high reactivity of HA and FA [174]. ACT has been mainly sorbed to HS fractions in soil; ACT has been sorbed in mineral particles, and Al^3+^ and Fe^3+^ ions through electrostatic interactions, but these play only a minor role in the ACT sorption process on soil under weakly acidic to neutral *pH* [174].

All of this evidence shows how difficult it is to control the environmental fate of pesticides (or even to be able to track their contamination levels in soil, water, or air). In order to assess how common it is for different non-target species, from urban, rural, agricultural, and adjacent wild habitats, to suffer from pesticide contamination, it is key to understand the probability with which different pesticides end in non-desired places. Thus, the study of the link between pesticide environmental fate and sublethal effects on non-target organism is a must. 

Sub-lethal effects on target pests are also an inevitable consequence of these applications. These exposures have been found to allow pests to develop resistance, for instance, through hermetic responses that have been also hypothesized to be linked with EMMs. This is developed further in the following section. 

## 4. Pesticide Effects on Pest and Non-Target Organisms

Pesticides may control target pest species, leading to a temporal/local decline in these non-desired organisms but can also lead to the development of resistance, among a wide range of other possible responses [3,43,122,123,176,177,178,179,180]. Such a range of responses has to do with a variety of factors, from the type of organism being exposed to the dose and venue of exposure [32]. For instance, under intensive agriculture management, pesticides are often reapplied several times due to their natural decrease in availability over time. It has been reported that pests can develop resistance to formulations during periods of exposure to lower pesticide concentration [10,44,181]. It has also been described that the use of pesticides may end up helping the pest they were intended to combat by suppressing pest predators and competitors that are naturally available in the ecosystem. Outbreaks of new pests that were under control before the application of the pesticide can emerge due to the development of resistance against pesticides by the pest species [3,43]. 

The exposure of non-target insects (such as parasitic wasps and social Hymenoptera) to sub-lethal levels of contamination may trigger several developmental and physiological alterations as well as may augment their susceptibility to develop diseases, thereby reducing their chances of survival [12,182]. The effects of pesticides on insects may depend, among other factors, on the mechanisms that the exposed organisms employ to process these toxicants [45]. These mechanisms include behavioral responses such as avoiding pesticides, metabolic adaptation to process toxic chemicals, epigenetic changes, and altered regulation of coding proteins [43,45,54,85]. 

Insects are recognized as the main collaborators in nature’s contribution to people, such as in food production [182]. For instance, data from USA show that the main crops produced are strongly dependent on insect pollination, where wild pollinators contribute to a large portion of the crop yield obtained [183]. This pattern is similar worldwide [184], even for crop species with autonomous self-reproduction [185]. This evidence stresses the need for understanding and considering the interaction of pesticides with pollinators. In the following section, we focus on neonicotinoid pesticides. Although these formulations were initially proposed as less harmful in comparison with other pesticide chemical formulations to non-target organisms such as insect pollinators, recent evidence demonstrates that this is not an accurate claim.

## 5. Neonicotinoid Insecticides

Neonicotinoids are third-generation broad spectrum pesticides intended for the control of sucking (e.g., aphids, whiteflies, leaf- and planthoppers, and thrips) and chewing pest insects (e.g., microlepidoptera and some Coleoptera) [123,186]. These pesticides can be translocated to different tissues as a systemic property of this type of pesticide; for instance, neonicotinoids can be absorbed by roots [41,187]. Neonicotinoids can also be applied by directly impregnating treated seeds, a method that transfers a great portion of the pesticides to the soil due to their high solubility in water [188]. The apparent benefits of neonicotinoid applications on plants have been overshadowed by recent studies showing that the use of thiamethoxam (hereafter “TMX”) and imidacloprid (hereafter “IMI”) on crop sunflowers *Helianthus annuus* L. (Asteraceae) induces mitotic phase irregularities as well as genotoxic effects (e.g., DNA damage and genome instability) [189]. Neonicotinoid pesticides have also been used in home gardens to control domestic pests and in cats and dogs to prevent fleas and ticks [190].

The most common active ingredients in neonicotinoids are IMI, acetamiprid, nitenpyram, TMX, thiacloprid, clothianidin (hereafter “CTD”), and dinotefuran [186]. However, novel formulations are continuously being produced. Neonicotinoid pesticides work by binding to the nicotine acetylcholine receptor (nAChR), thereby affecting the central nervous system of insects [118,190,191]. As acetylcholine receptors were proposed as less important for vertebrates than invertebrates, neonicotinoids as pesticides were promoted to be safer for non-target organisms (such as humans) [187]. These ideas have promoted an indiscriminate use of these pesticides in all types of crops and soils, even though the human health impacts of exposure to these chemicals is not fully understood [187].

Often, pesticide application methods, both agricultural and domiciliary, do not appropriately discriminate between target and non-target organisms. Neonicotinoids are not an exception to this problem [192,193,194]; many different organisms (e.g., insects) may be affected by these toxicants, triggering varied responses [44,195,196]. Neonicotinoid contamination of non-target organisms has been extensively documented in animals [197,198,199]. For example, neonicotinoid contamination can impact native fauna such as the Japanese crested ibis, where exposure to CTD was found to be detrimental to reproduction, most likely due to triggering oxidative stress [197]. IMI can alter early development in mammals (e.g., mice). These alterations were found to be related to C5-cytosine methylation due to exposure to this neonicotinoid pesticide [200]. Exposure to acetamiprid has been found to be linked to DNAm in rat brain and liver [201]. There have been studies in mice showing that neonicotinoid and acetamiprid can cross the blood–brain barrier, which highlights the severity of the potential risks to mammals [166]. Thus, neonicotinoids trigger alterations in different regulatory process, including EMMs, in vertebrates. 

Varied sub-lethal detrimental effects and epigenetic consequences have been also found in other life forms. It has been demonstrated that neonicotinoids are extremely toxic to a great diversity of invertebrates. For instance, the evaluation of the impact of the IMI-containing insecticide “Tree and Shrub”™ on wildtype *Caenorhabditis elegans* (Maupas, 1900) (Nematoda: Rhabditidae) disrupted fertility along with growth and locomotion [202]. It has been demonstrated in Gastropoda that neonicotinoid thiacloprid damages the central nervous system of the native species *Lymnaea stagnalis* (Linnaeus, 1758) (Hygrophila: Lymnaeidae), the great pond snail, through its modulation of nicotinergic acetylcholine receptors, hampering cholinergic neurotransmission [203].

Neonicotinoids lead to death and to a wide array of critical sub-lethal impacts in Insecta evaluated in field-realistic exposure, including alterations linked to EMMs (both in managed and wild species) [43,119,137,158,204]. These consequences led to the loss of the ecosystem services provided by these insects, including nutrient cycling and pollination [205]. These declines in local insect biodiversity have been shown to be linked to unexpected costs in agricultural production under industrialized management and impacts on the health of managed agroecosystems [206]. Although non-target insects may be exposed to lower doses of insecticides than target ones, for flying insects, this situation depends also on chance, unless they exhibit behavioral resistance [45]. Exposure to lower doses might not kill them, but there is recent evidence of their impact on different regulatory processes at different scales [140], including providing the descendants with resistance to this pesticide [83,207] and increasing their population via hormetic response [182,208]. In insects, this has been chiefly studied in managed bees *Apis mellifera* (Linnaeus, 1758) [187]. Nonetheless, there is also a growing body of evidence demonstrating adverse consequences on other managed as well as native insects [209,210,211].

For instance, in the parasitic wasps key for biological control in agricultural management such as *Nasonia vitripennis* (Walker, 1836) (Hymenoptera: Pteromalidae), the neonicotinoid imidacloprid disrupts sex allocation cues, reducing their reproductive success [212]. Sub-lethal IMI exposure in the Colorado potato beetle, pest *Leptinotarsa decemlineata* Say, 1824 (Coleoptera: Chrysomelidae) has been linked to alterations in specific genes, C5-cytosine methylation, and transposable elements that have been postulated as linked to the development of insecticide resistance [81].

Similar patterns were found for one of the major pests of fruit orchards: the codling moth *Cydia pomonella* (Linnaeus, 1758) (Lepidoptera: Tortricidae). In this pest species, it was found that acetamiprid exposure in a field population triggered a significant increase in biotransformation and antioxidant enzyme activity; the detoxification of acetamiprid was proposed to be achieved by methylation [213]. These hormesis-like resistance patterns were proposed by the authors to be linked with epigenetic mechanisms [213,214].

These consequences on insect biology of the exposure to these toxicants are very likely to be shared between most studied insects (e.g., managed species and model insects) and native insects. For instance, EMMs found to be involved in alterations detected in managed bees due to the application of neonicotinoid pesticides may also impact wild bee species and other native insect species, especially in agricultural landscapes [215]. These plausible scenarios are discussed in the following section, with a particular focus on bees.

### Neonicotinoid Effects on Bees

There is an abundant body of evidence that shows that sub-lethal doses of neonicotinoids have direct effects on several vital functions of *Apis mellifera* [41,48,83,120,187,216], from reproductive ability to cognitive impairments. These issues have been linked to epigenetic mechanisms [45]. For instance, different authors have described changes in histone acetylation and deacetylation [120,215], their transgenerational inheritance [76], and C5-cytosine methylation effects [41].

There has been strong evidence for almost a whole decade on the involvement of sub-lethal doses of neonicotinoids in the development of colony collapse disorder (hereafter “CCD”). Of particular interest is an experiment showing that the use of high-fructose corn syrup (HFCS) as an alternative to honey or sucrose-based food was related to CCD [48]. When the authors investigated the composition of the HFCS used, they found sub-lethal dosages of imidacloprid, a neonicotinoid insecticide widely used in the Americas (Canada, the United States, the Caribbean, and Central and South American countries) and China [119,190,207,217,218]. As previously mentioned, imidacloprid and other neonicotinoids are systemic pesticides applied directly to seeds through impregnation, which stay on the plant during its development and are detected in most plant tissues [187], and might end up in the final product that humans and other animals consume, although in much lower concentrations than the original dose [219].

The effects of neonicotinoids on bees are many and wide-ranging (e.g., [137]), including decreased microglomerular density of mushroom bodies [220], symptoms of neurotoxicity [191], reduced fecundity in queens and males [41], and impaired immune response [221], among other issues that challenge the sustainability and survival of colonies [43]. Memory issues in bees have being linked to epigenetic changes, specifically in relation to histone acetylation [120] and DNAm [222]. There is also evidence that exposure to neonicotinoids has global DNA methylation effects on honeybees [119].

Evidence suggests that CCD is related to the emergence of neonicotinoids and their massive use in agriculture [120]. This would be mainly due to the previously described sub-lethal effects of pesticides [41,43,221], which impair the proper functioning of colonies, leading to their ultimate collapse [48,216,220,223].

Ongoing studies are being conducted to understand how to counteract the detrimental effects of neonicotinoids, with sodium butyrate as a possible candidate antidote to the epigenetic modifications experienced by bees exposed to neonicotinoids [120,216]. This kind of research is of high importance due to the menace that declining numbers of bees and other flying insects represent for all kinds of ecosystems and for human survival [15,224]. Despite all of these problems detected in different non-target organisms, the use of neonicotinoids is currently poorly regulated, especially in developing countries [205].

## 6. The Link between Insect EMMs and Current Loss of Biodiversity

Organisms around the globe are currently experiencing emerging pressures that threaten their survival and are causing the extinction of an unprecedented number of species in a short span of time [28,30]. Insects are being seriously affected by these pressures and their numbers are declining quickly [15], while we can hardly quantify the losses due to the incompleteness of the available information [176].

The main drivers of the global environmental crisis have been classified as “planetary boundaries” or safe operational limits within which the perpetuation of humanity is feasible [225]. Global biodiversity decline is one of the most worrisome limits [224]. The loss of biodiversity has been linked to other planetary boundaries such as climate change, pollution, alteration of biogeochemical cycles, and habitat loss and degradation [45,89]. This last means that habitat quality decrease, for instance, due to fragmentation (i.e., increasing habitat isolation) [29]. These drivers are deeply interdependent and are the main reasons behind the visible reduction in biodiversity that affects our planet. Their interactions and effects are of utmost scientific interest, since most species are threatened by a combination of these and other planetary boundaries instead of the alteration of a single one [224].

The increased weather volatility brought about by climate change [226] and its effects are now felt in a wide variety of ecosystems [31,227,228]. There is evidence from previous extinction events that high-frequency fluctuations in mean temperatures and even in geological timescales may elevate the extinction rate in marine invertebrates compared with more stable periods [229]. Thus, the effects of climate change and increasing temperatures in both the upper and deep ocean [230] might have catastrophic results not only for marine biodiversity but also on terrestrial processes highly dependent on its stability [225].

Pollution directly decimates biodiversity not only due to its toxic effects on living organisms but also through the contamination of non-renewable natural resources such as soil and water. Pollution derived from the human productive industry has been stressed as the base of biodiversity loss [174,231]. These alterations are also linked to the dramatic alteration of biogeochemical cycles, mostly mediated by soil microorganisms and associated invertebrates [225]. Contamination leads to habitat loss, which is closely related to anthropogenic activities such as intensive agriculture, urban development, and other extractive activities that humans perform in order to thrive under the current economic paradigm [232]. Pesticides are an important factor in habitat loss, which affects the biology of non-target organisms [83,93,98,111,117,122] and/or behavior [105,116,127,233], with potentially lethal consequences [107,108,114].

Habitat fragmentation is caused by a wide array of anthropogenic activities such as intensive agricultural practices [29], with monocultures in huge portions of land affecting whole ecosystems [234], the process of urbanization [235,236] with the building of roads to connect cities that slash through habitats [237,238,239], and land and sea exploitation for the extraction of fossil fuels or minerals [240,241].

The decrease in habitat quality is deeply linked to the contamination derived from human activities [242,243] such as soil degradation by intensive agriculture and subsequent land abandonment [244,245]; extensive use of fertilizers and the associated nitrogen accumulation [29]; indiscriminate application of pesticides with direct and indirect effects on trophic chains [122,178,180,246]; pollution of soil [151,247], and nearby water bodies and streams [93,98,124,188]; road building [237,238]; and inappropriate management of protected habitats [248].

The abovementioned drivers affect the resilience of the different ecosystems that are exposed to them, reducing the chances of recovery of biodiversity and its functional relations [29]. Different organisms react in a wide array of ways in response to these drivers. While some organisms perish, others are able to adapt through different strategies, which include EMMs [80,249]. In fact, EMMs may be key to the survival of entire species because they allow for fast responses to a changing environment, which may be fundamental for the survival of organisms [80,250].

As shown in Table 2, many studies have been performed in artificial [84,89,101,125] and natural [91,94,122] aquatic settings to emulate scenarios of water pollution. This kind of study is vital in order to gauge the impact of the presence and application of chemicals in the environmEntomol. Since aquatic insects are routinely used as bioindicators of the toxicity of different chemicals [98], it is not surprising that interest in their epigenetic responses to harmful stimuli has grown recently [11,84,85].

As previously mentioned, the response of an insect to pesticide exposure depends on the amount of chemical involved [83,134]. The chemical behavior of pesticides in the environment as well as their inaccurate application often lead to the development of resistance in target organisms or even to hormetic responses [115,116,208]. These adaptive mechanisms include epigenetic changes that allow organisms to modify biological processes via changes in gene expression [83]. It has been proposed that new formulations of pesticides should consider their potential epigenetic effects in order to increase their effectiveness and to reduce the damage to non-target species [44,45,80]. The use of insect EMMs has also been proposed as a promising venue for the development of methods to ameliorate the negative effects of pesticides once considered “safe”. One example of this approach is the application of histone deacetylase inhibition treatment in order to restore learning abilities in honey bees with memory impairment caused by neonicotinoid exposure [120].

While the effects of pesticides on non-target organisms have been widely studied [148], to our knowledge, a direct comparison between a native versus an introduced species in order to assess whether there are differences in their responses towards a specific pesticide has not been made (Table 2). A few studies have been conducted in bees [251] and ants [252]. In these evaluations, contrasts have been shown on how pesticides affect native and introduced species, suggesting that while native species suffer mostly detrimental effects, pests are capable of developing resistance with sub-lethal exposures. These trends stress the need for further research on this subject, especially the relation between pesticides and EMMs, as these links may hold the key for these contrasting responses to stress derived from agrochemicals. This would allow for better understanding of the potential damage that pesticides may inflict in ecosystems and helps manufacturers produce more precise pesticides that inflict less environmental damage [253].

We must reiterate the importance of investigating EMMs involved in the responses of insects towards pesticide exposure. As shown in Table 1, to the best of our knowledge, there are at least three main processes described as EMMs. Thus far, insect EMMs triggered in relation to the exposure of environmental toxicants are mainly DNAm and RNA-based mechanisms, while in vertebrates, it has been shown that histone alterations can also be triggered due to pesticide exposure. This has been found in rats exposed to the pesticide Dichlorodiphenyltrichloroethane, DDT [254]. These differences may be due to the nature of these particular EMMs and the biases towards the study of vertebrates as a proxy for human health. Nonetheless, considering that the abovementioned evidences showing that histone deacetylase inhibitors are capable of upregulating memory-related genes in IMI memory-damaged bees [119], it is possible to suggest a key role of post-translational processes in relation to insect epigenetic responses facing the exposure to toxicant stress. Despite this, it is also probable that there is a lack of studies on other species besides canonical model organisms and ecological contexts involved in insect epigenetic responses towards toxicants. This scenario emphasizes the need for further research on this topic both from applied and basic points of view.

The actual complexity of the relationship between inheritance, environmental cues, and their phenotypic consequences found in different systems (including insects) allows us to recognize that EMMs contribute to the systemic regulation and stabilization properties of living organisms [255]. Understanding the biological mechanisms involved in insect epigenetic responses towards agrochemicals, what stage of insect ontogeny it affects, and what type of disturbances or compensations are triggered in its features is fundamental to assessing the impact of pesticides in insects (native, managed, and pest species). This may hold the key to reducing the damage of pesticides to non-target species and thus helps in the fight against the current global decline in biodiversity [45,83].

## 7. Conclusions

In a rapidly changing world with several planetary boundaries near collapse [225], the consideration of the role of EMMs in the context of insect exposure to variated and abundant human-derived stressors (such as pesticides) may be relevant to prevent further population declines in wild insect species. This understanding also helps to prevent the development of pesticide resistance in pests and introduced species that should be controlled [83]. Intensified schemes of agricultural production have been shown to be the main contributors to biodiversity decline globally, including the demise of pollinating insects [256,257,258,259]. In particular, pesticide use is one of the main factors responsible for the decimation of managed as well as native pollinators [135,157,260].

By studying insect EMMs in response to stressors both in the wild and in agriculturally managed ecosystems, it becomes possible to understand why some species can adapt rapidly while others perish. As we have shown here, there is strong evidence that supports the thesis that responses involving EMMs should be considered in the formulation and testing of new pesticides as well as in the search for more sustainable and ecologically minded alternatives. These new developments may be fundamental to understanding the biological responses of affected organisms to control measures and may provide tools to avoid formulations that have proven detrimental effects on ecosystems [44,45,80]. New knowledge and technology developed based on EMMs in insects may allow us to find ways to ameliorate the consequences of pollinator exposure to pesticides. Promising results have been shown in honeybee memory repair through treatments involving EMMs [120]. A better understanding of EMMs in insects could be an important resource in the fight against the loss of biodiversity that we currently face in what has been called the sixth mass extinction [28,32,240,261].

## 8. Further Remarks

The epigenetic effects found in insects due to pesticide exposure should be “the canary in the coal mine” for the consequences of contamination on our own species and a source of development for novel technologies to allow us to have a more sustainable relationship with biodiversity. The chemical behavior of different pesticides demonstrates that it is almost impossible to control the contamination of soil and water sources, and the exposure of non-target organisms to the current toxic formulations [161,165,171]. This pollution may affect vulnerable native and endemic species, where EMMs are found to be associated with stress and abnormal functioning in insects. To worsen the situation, among non-target organisms, it is possible to find undesired pest and invasive insects, where EMMs lead to the development of tolerance and resistance against pesticides. There is evidence for the role of EMMs in the development of resistance towards pesticides. This has been demonstrated in insects with aquatic immature stages such as mosquitoes [11].

Thus, a change towards a better understanding of insect epigenetics as well as the application of agroecological approaches promoting sustainable agriculture is a must [54,205,262]). Future evidence of EMM-related changes in insects exposed to pesticides may serve as bioindicators of potentially dangerous levels of exposure to contamination in our own species. This is another promising venue of research in relation to EMMs in insects and their effects in response to anthropogenically derived contamination.

## Figures and Tables

**Table 1:** Currently described mechanisms of stable epigenetic inheritance *.

Epigenetic Inheritance Mechanism	Heritable Effect	Reference
C5-cytosine methylation/demethylation	Phase variation	Pearson (2019) [17]
	Inheritance of methylated *Cori* *	Frandi & Collier (2019) [18]
	Epimutations	Skinner et al. (2019) [19]
	Paramutations	House & Lukens (2019) [20]
	Genomic imprinting	Tucci et al. (2019) [21]
	Transcriptional silencing	Di Felice et al. (2019) [22]
	X chromosome inactivation	Żylicz et al. (2019) [23]
Histone modifications	Dosage compensation	Shevchenko et al. (2019) [24]
	Vernalization	Zhong et al. (2019) [25]
Post-transcriptional silencing through RNA interference	Transgenerational inheritance of neural processes	Posner et al. (2019) [26]
	Heritable effects of starvation	Dupont et al. (2019) [27]

* Chromosome replication origin.

**Table 2:** Responses of insects to different toxicants. The first column indicates the kind of chemical compound described; the second indicates the compound. The third lists the affected species. The fourth column indicates the order and family of the insect. The fifth lists the effects observed in response to the toxicant. The sixth shows the epigenetic molecular mechanisms involved, where “DNA M.” refers to C5-cytosine methylation, “H. M.” stands for histone modifications, and “RNA-b M.” is RNA-based mechanisms [54]. The seventh column has the corresponding references cited.

Chemical Group/ Functional Category	Elements or Compounds	Affected Species	Order: Family	Effects	EMMs Studied	References
Heavy Metals	Cu^2+^	*Aedes aegypti; Anopheles arabiensis*	Diptera: Culicidae	Cell metabolisms, egg hatching, apoptosis; decrease in RNA methylation; DNAm methylation	DNA M., RNA-b M	Rayms-Keller et al., 2000 [89]; Raes et al., 2000 [90]; Jeanrenaud, Brooke & Oliver, 2020 [84]
	Zn	*Rhithrogena robusta*	Ephemeroptera: Heptageniidae	Reduced individual growth rate	NO	Carlisle & Clements, 2003 [91]
	Pb	*Lymantria dispar; Anopheles arabiensis*	Lepidoptera: Erebidae; Diptera: Culicidae	Decrease in growth and reduction in hatching success; increased in RNA methylation patterns; DNA methylation	DNA M., RNA-b M.	Gintenreiter, Ortel & Nopp, 1993 [87]; Jeanrenaud, Brooke & Oliver, 2020 [84]
	Cd	*Orchesella cincta; Anopheles arabiensis*	Collembola: Entomobryidae; Diptera: Culicidae	Transcriptome stress response; increase in 5-hmC methylation	DNA M.	Roelofs et al., 2009 [86]; Jeanrenaud, Brooke & Oliver, 2020 [84]
Heavy Metals	Al	*Drosophila melanogaster*	Diptera: Drosophilidae	Reduction in median life span; climbing ability and cognitive capacity	NO	Wu et al., 2012 [92]
Chlorinated hydrocarbons	DDT	*Ephemerella subvaria, Ephemerella auravillii*	Ephemeroptera: Ephemerellidae	Impaired subimago emergence	NO	Hitchcock, 1965 [93]
	Aldrin	*Cheumatopsyche analis*	Trichoptera: Hydropsychidae	Hormetic response	NO	Moye & Luckmann, 1964 [94]
	Chlordane	*Periplaneta americana*	Blattodea: Blattidae	Increase in total hemocyte count; excessive vacuolization of epithelial cells in the midgut lumen	NO	Gupta & Sutherland, 1968 [95]
	Endrin	*Periplaneta americana*	Blattodea: Blattidae	Dose-dependent blocking of GABA receptors	NO	Wafford et al., 1989 [96]
Chlorinated hydrocarbons	Heptachlor	*Periplaneta americana*	Blattodea: Blattidae	Dose-dependent blocking of GABA receptors	NO	Lummis et al., 1990 [97]
	Lindane	*Periplaneta americana; Chironomus ripariu*	Blattodea: Blattidae; Diptera: Chironimidae	Dose-dependent blocking of GABA receptors; reduction in imago emergence	NO	Wafford et al., 1989 [96]; Maund et al., 1992 [98]
Organophosphates	Parathion	*Musca domestica*	Diptera: Muscidae	Toxin degradation	NO	Matsumura & Hogendijk, 1964 [99]
	Malathion	*Periplaneta americana; Musca domestica*	Blattodea: Blattidae; Diptera: Muscidae	Toxin metabolization	NO	Krueger & O’Brien, 1959 [100]
	Glyphosate	*Deleatidium spp.*	Ephemeroptera: Leptophlebiidae	Reduction in imago emergence	NO	Magbanua et al., 2016 [101]
Organophosphates	Diazinon	*Musca domestica*	Diptera: Muscidae	Toxin degradation	NO	Matsumura & Hogendijk, 1964 [99]
	Tetrachlorvinphos	*Alphitobius diaperinus*	Coleoptera: Tenebrionidae	Resistance to pesticide	NO	Hamm et al., 2006 [102]
	Azamethiphos	*Musca domestica*	Diptera: Muscidae	Resistance to pesticide	NO	Kristensen et al., 2000 [103]
	Phosmet	*Megachile rotundata*	Hymenoptera: Megachilidae	Reduced nesting and progeny production	NO	Alston et al., 2007 [104]
	Diclorvos	*Alphitobius diaperinus*	Coleoptera: Tenebrionidae	Resistance to pesticide	NO	Chernaki-Leffer et al., 2011 [105]
	Terbufos	*Alphitobius diaperinus*	Diptera: Sarcophagidae	Avoidance of pesticide	NO	Jales et al., 2020 [106]
Carbamates	Sevin	*Musca domestica*	Diptera: Muscidae	Metabolization of pesticide	NO	Eldefrawi & Hoskims, 1964 [107]
	Aldicarb	*Pseudatomoscelis seriatus; Musca domestica*	Hemiptera: Miridae; Diptera: Muscidae	Death	NO	Davis & Cowan, 1972 [108]; Spurr & Sousa, 1974 [109]
	Carbofuran	*Diabrotica virgifera*	Coleoptera: Chrysomelidae	Increase in oviposition; increase in longevity	NO	Ball & Su, 1979 [110]
	Carbaryl	*Diabrotica virgifera*	Coleoptera: Chrysomelidae	Increase in oviposition	NO	Ball & Su, 1979 [110]
Pyrethroids	Allethrin	*Periplaneta americana*	Blattodea: Blattidae	Temperature dependent disruption of the nervous system	NO	Gammon, 1978 [111]
	Bifenthrin	*Apis mellifera ligustica*	Hymenoptera: Apidae	Reduction in oviposition; reduction in cap rate; reduction in emergence rate; success rate of development	NO	Dai et al., 2010 [112]
	β Cyfluthrin	*Drosophila melanogaster* (Sepia mutant)	Diptera: Drosophilidae	Reduction imago emergence; prolongation of total developmental period	NO	Nadda, Saxena & Srivastava, 2005 [113]
	Cypermethrin	*Alphitobius diaperinus*	Coleoptera: Tenebrionidae	Resistance to pesticide	NO	Chernaki-Leffer et al., 2011 [105]
	Cyphenothrin	*Ranatra filiformis*	Hemiptera: Nepidae	Hyperactivity, death	NO	Saha & Kaviraj, 2007 [114]
Pyrethroids	Deltamethrin	*Apis mellifera ligustica*	Hymenoptera: Apidae	Reduction in oviposition; lower hatch rate; reduction in cap rate; success rate of development	NO	Dai et al., 2010 [112]
	Permethrin	*Acheta domesticus*	Orthoptera: Gryllidae	Death	NO	Schleier & Peterson, 2010 [115]
	Resmethrin	*Danaus plexippus*	Lepidoptera: Nymphalidae	Reduced adult size, death	NO	Oberhauser et al., 2009 [116]
	Transfluthrin	*Culex tarsalis*	Diptera: Culicidae	Avoidance of pesticide	NO	Britch et al., 2020 [117]
Neonicotinoids (neuroinsecticides)	Thiamethoxam	*Musca domestica*	Diptera: Muscidae	Acetylcholine receptors hyperexcitation; ATPase activity	NO	Abdel-Haleem et al., 2018 [118]
Neonicotinoids (neuroinsecticides)	Imidacloprid	*Apis mellifera*	Hymenoptera: Apidae	Acetylcholine receptors hyperexcitation; Malpighian tubule deformation; changes in global DNA methylation	DNA M.	Paleolog et al., 2020 [119]; Hu et al., 2018 [120]; Brevik et al., 2020 [81]
	Acetamiprid	*Apis mellifera*	Hymenoptera: Apidae	Reduction in sucrose sensitivity; increased locomotive activity	NO	El-hassani et al., 2007 [121]
	Clothianidin	*Chironomus dilutus*	Diptera: Chironomidae	Reduction of emergence	NO	Maloney et al., 2018 [122]
	Nitenpyram	*Bemisia tabaci B biotype*	Hemiptera: Aleyrodidae	Resistance to pesticide	NO	Liang et al., 2012 [123]
	Thiacloprid	*Culex pipiens*	Diptera: Culicidae	Preimaginal development duration	NO	Beketov & Liess, 2008 [124]
	Dinotefuran	*Chironominae spp.*	Diptera: Chironomidae	Population hormetic response	NO	Kobashi et al., 2017 [125]
Endocrine disruptors (ED)	Bisphenol A	*Chironomus riparius*	Diptera: Chironomidae	Increase in mRNA for ecdysone receptor and increase in the expression of HSP70	RNA-b M.	Planelló, Martínez-Guitarte & Morcillo, 2008 [85]
	Tributyltin	*Chironomus riparius*	Diptera: Chironomidae	DNA breakage	NO	Martínez-Paz et al., 2013 [126]
	Pentachlorophenol	*Chironomus riparius*	Diptera: Chironomidae	Upregulation of Hsp70 gene transcription; downregulation of the Hsp27 transcription	NO	Morales et al., 2014 [127]
	Nonylphenol	*Chironomus riparius*	Diptera: Chironomidae	DNA breakage	NO	Martínez-Paz et al., 2013 [126]
	Triclosan	*Chironomus riparius*	Diptera: Chironomidae	DNA breakage	NO	Martínez-Paz et al., 2013 [126]
	Benzyl butyl phthalate	*Chironomus riparius*	Diptera: Chironomidae	Overexpression of the EcR gene	NO	Planelló et al., 2011 [128]
Endocrine disruptors (ED)	DEHP/Di(2-ethylhexyl)phthalate	*Chironomus riparius*	Diptera: Chironomidae	Mouthparts deformities	NO	Park & Kwak, 2008 [129]
	Ethinylestradiol	*Drosophila melanogaster*	Diptera: Drosophilidae	Reduction in lifespan, decrease in fertility	NO	Bovier et al., 2018 [130]
	Genistein/5,7-dihydroxy-3-(4-hydro- xyphenyl)chromen-4-one)	*Aedes albopictus*; *Anopheles arabiensis*	Diptera: Culicidae	DNA methylation; reduction in egg hatching	DNA M.	Oppold et al., 2015 [11]; Jeanrenaud, Brooke & Oliver, 2020 [84]
	Vinclozolin (Fungicide)	*Aedes albopictus*; *Anopheles arabiensis*	Diptera: Culicidae	DNA methylation; reduction in egg hatching	DNA M.	Oppold et al., 2015 [11]; Jeanrenaud, Brooke & Oliver, 2020 [84]
	DMSO/dimethyl sulphoxide	*Antheraea assamensis*	Lepidoptera: Saturniidae	Alterations in hormonal balance; alterations in silk production	NO	Unni et al., 2009 [131]
Micro plastics	Polystyrene	*Culex pipiens*	Diptera: Culicidae	Accumulation in Malpighian tubules	NO	Al-Jaibachi, Cuthbert & Callaghan, 2018 [132]
	Polypropylene	*Lestes viridis*	Odonata: Lestidae	Accumulation in body	NO	Akindele, Ehlers & Koop, 2020 [133]
	Acrylonitrile butadiene styrene (ABS)	*Siphlonurus* sp.	Ephemeroptera: Siphlonuridae	Accumulation in body	NO	Akindele, Ehlers & Koop, 2020 [133]

## Data Availability

All data are presented in the manuscript.
